# A demonstration of the susceptibility of clinical isolates obtained from cystic fibrosis patients to manuka honey

**DOI:** 10.1007/s00203-015-1091-6

**Published:** 2015-02-14

**Authors:** Rowena Jenkins, Mandy Wootton, Robin Howe, Rose Cooper

**Affiliations:** 1 Department of Biomedical Sciences, Cardiff School of Health Sciences, Cardiff Metropolitan University, Western Avenue, Cardiff, CF5 2YB Wales, UK; 2 Public Health Wales, Microbiology Cardiff, University Hospital of Wales, Heath Park, Cardiff, CF14 4XW Wales, UK

**Keywords:** Cystic fibrosis, *Pseudomonas aeruginosa*, *Burkholderia cepacia*, Manuka honey, Synergy

## Abstract

*Pseudomonas* and *Burkholderia* pose a significant health threat to people with chronic respiratory conditions; the resistance inherent in these bacteria indicates that new antimicrobial strategies are required. Susceptibility of 56 strains of *P. aeruginosa* and 55 strains of *Burkholderia* to manuka honey, tobramycin and colistin using microbroth dilution and E strip was determined. MICs of antibiotics with honey were determined to search for synergistic combinations against two representative strains of each genus. All strains exhibited susceptibility to honey ≤10 % (w/v); mean susceptibility of *Burkholderia* (4.6 % w/v) was lower than *P. aeruginosa* (7.3 % w/v). Synergistic or additive combinations were found with all four strains tested. Combinations of manuka honey with antibiotics can be used to lower the MIC need to successfully inhibit both *P. aeruginosa* and *B. cepacia*. The use of honey as a combination agent may be possible for the management of *P. aeruginosa* and *B. cepacia.*

## Introduction

*Pseudomonas aeruginosa* and *Burkholderia* spp. are important opportunist bacteria that can cause serious and chronic respiratory infections in vulnerable patients, especially those with underlying conditions such as cystic fibrosis or chronic granulomatous disease (Lyczak et al. 2000; Vanlaere et al. 2009). They have also been implicated in wound infections (Alvarez-Lerma et al. 2008; Bassett et al. 1970). The switch from non-mucoid to mucoid variants accompanies complex changes in motility and biofilm formation that have been shown to facilitate the persistence of each of these bacteria in lungs. Chronic colonisation of the lungs of cystic fibrosis patients by mucoid strains of *P. aeruginosa* and *Burkholderia* is associated with deteriorating lung function which results in a poor prognosis (Hancock 1998; Nicas and Hancock 1983).

Increasingly, the tendency of pathogenic bacteria to acquire resistance to conventional antibiotics is limiting treatment plans, and new strategies of antimicrobial treatment are being sought. In comparison with other bacteria, the low permeability of the outer membrane of *P. aeruginosa* restricts penetration of antibiotics into the bacterium (Hancock 1998); this membrane is 12–100 times less permeable than that of *E. coli* (Nicas and Hancock 1983). As a result of this intrinsic mechanism, *P. aeruginosa* can exhibit high baseline decreased susceptibility to many antibiotics. Increasingly, gene mutation and gene acquisition confer resistance to a range of antibiotics that includes β-lactams, aminoglycosides and fluoroquinolones, making these strains more difficult to inhibit.

*Burkholderia* strains are also inherently resistant to many available antibiotics (Mahenthiralingam et al. 2005). It has been demonstrated that some strains utilise penicillin as a sole carbon source (Vermis et al. 2003) and that others survive in solutions of the hospital disinfectant, chlorhexidine (Heo et al. 2008). Some of this recalcitrance may be due to their wide distribution throughout the environment; they have been isolated from soil, water, insects and plants, as well as from humans, where they will have been exposed to many antimicrobial compounds (Compant et al. 2008).

Both of these notoriously resistant bacteria are prolific biofilm formers, especially in cystic fibrosis patients where up to 1000 times the concentration of antibiotic is needed than to kill the equivalent planktonic cell (Mah and O’Toole 2001). Added to the natural resistance of these bacteria, this makes these tenacious bacteria a real therapeutic challenge. New strategies are needed to treat for cystic fibrosis pulmonary infection (McCaughey et al. 2013).

Susceptibility to manuka honey of 22 strains of *B. cepacia* (Cooper et al. 2000) and of 17 strains of *P. aeruginosa* isolated from burns (Cooper et al. 2002) has been demonstrated using raw non-medical-grade (non-sterilised) honey samples. Gamma-irradiated sterile medical-grade honey is now available, and it is this which is now utilised for both medical research and in licensed wound dressings applied within wound care. Recently, manuka honey has been shown to enhance the activity of antibiotics (Jenkins and Cooper 2010, 2012; Muller et al. 2013). Currently, the susceptibility to manuka honey of clinical strains of *P. aeruginosa* and *B. cepacia* isolated from cystic fibrosis patients and the potential for synergistic activity in combinations of antibiotics and honey against these strains is unknown. This study, therefore, was designed to determine the susceptibility of clinical isolates to medical-grade honey and also to look at the potential for honey in combination with antibiotics used in this field.

## Materials and methods

### Test bacteria and honey

Clinical isolates of *P. aeruginosa* and *B. cepacia* collected from a range of infections were submitted to the Specialist Antimicrobial Chemotherapy Unit, Cardiff in the UK. Of the isolates tested here, 56 were identified as *P. aeruginosa* and 55 as *Burkholderia* species by standard bacteriological techniques (Table 1). These cultures were stored in −80 °C on beads and cultured on Columbia agar containing horse blood before testing.

**Table 1:** Minimum inhibitory concentrations of manuka honey (% w/v) against clinical strains of *Burkholderia* and *Pseudomonas aeruginosa* were determined by the microbroth dilution method

Strain	Genomovar	Number	Mean MIC (% w/v)	SD	Range (% w/v)
*B. cepacia complex*	I	4	4	0.8	3–5
*B. cepacia*	I	6	5.2	1.6	3–7
*B. cepacia group K*	I	3	4.3	0.6	4–5
*B. multivorans*	II	10	4.6	1.1	3–6
*B. cenocepacia*	III	15	4.5	1.1	3–6
*B. cenocepacia*	III	1	6	0	6
*B. stabilis*	IV	1	5	0	5
*B. vietnamensis*	V	5	4.8	0.8	4–6
*B. ambifaria*	VII	4	4.5	0.6	4–5
*B. anthina*	VIII	4	4.25	1.3	3–6
*B. pyrrocinia*	IX	2	5	1.4	4–6
*P. aeruginosa*		56	7.3	2.0	3–10

The sterile medical-grade manuka honey used here was Comvita manukacare 18+ ; it was a gift from Comvita UK.

### Microbroth dilution

The minimum inhibitory concentration for manuka honey, colistin and tobramycin was determined by using the standard CLSI broth microdilution method with Mueller–Hinton broth (MHB). A serial doubling dilution was used for the antibiotics, but the MIC of the sample of honey was used at 1 % (w/v) increments from 0 to 10 % (w/v). Inocula were prepared from direct colony suspension, and microtitre plates were inoculated with 10^5^ CFU/ml. Plates were incubated in air at 37 °C for 18 h. The MIC was defined as the lowest concentration of the antimicrobial that prevents visible growth of a microorganism in a broth dilution susceptibility test.

### Synergy

Experiments were undertaken to determine the potential of honey in combination with currently used antibiotics to exhibit synergistic activity. Two representative isolates of each of *P. aeruginosa* and *B. cepacia* were chosen for this further investigation, and interactions between manuka honey and each of two antibiotics (colistin and tobramycin) were tested. Microbroth dilution was conducted as above using honey at 1 % (w/v) below the MIC with a range of antibiotic concentrations. These combinations were also tested by E strips by the inclusion of honey at 1 % (w/v) below the MIC into Muller Hinton agar (MHA) or MHA alone.

Fractional inhibition concentration (FIC) was calculated for each combination using the following formula:

$$ \begin{aligned} {\text{FIC}}\;{\text{of}}\;{\text{drug}}\;{\text{A}} = {\text{MIC}}\;{\text{of}}\;{\text{agent}}\;{\text{A}}\;{\text{in}}\;{\text{combination/MIC}}\;{\text{of}}\;{\text{agent}}\;{\text{A}}\;{\text{alone}} \\ {\text{FIC}}\;{\text{of}}\;{\text{drug}}\;{\text{B}} = {\text{MIC}}\;{\text{of}}\;{\text{agent}}\;{\text{B}}\;{\text{in}}\;{\text{combination/MIC}}\;{\text{of}}\;{\text{agent}}\;{\text{B}}\;{\text{alone}} .\\ \end{aligned} $$

The FIC values have been interpreted to indicate synergism (≤0.5), additivity (>0.5 to ≤4) or antagonism (>4) according to published recommendations (Carmeli et al. 1999; Odds 2003).

## Results

### Susceptibility of respiratory isolates to manuka honey

Using the standard CLSI microbroth dilution method, susceptibility to manuka honey was determined for 111 clinical isolates of *P. aeruginosa* and *B. cepacia* obtained from cystic fibrosis patients. The 56 isolates of *P. aeruginosa* exhibited an MIC of ≤10 % (w/v), and the 55 *Burkholderia* isolates exhibited an MIC of ≤7 % (w/v) (Table 1); mean MICs were 7.3 and 4.7 % (w/v) for *P. aeruginosa* and *B. cepacia,* respectively. Hence, *B. cepacia* demonstrated a higher susceptibility to manuka honey than *P. aeruginosa*; conversely, *B. cepacia* has been shown to be more resistant to antibiotics than *P. aeruginosa* (Wilkinson and Pitt 1995).

### Synergy between manuka honey and antibiotics

Using two representative strains of each of *P. aeruginosa* and *B. cepacia*, synergistic combinations of honey and either colistin or tobramycin were investigated using broth dilution and E strips. No antagonism was seen with any of the combinations tested. Synergy was established between colistin and honey for both *P. aeruginosa* isolates (Tables 2) and one of the *B. cepacia* isolates (Table 3). Synergy was also observed for tobramycin and honey against one of the *P. aeruginosa* species. All other combinations showed additivity with values ≤1. Despite some variation between MICs derived by different methods, FIC values were largely consistent (Tables 2, 3) .

**Table 2:** Susceptibility and FIC values of two clinical cystic fibrosis isolates of *P. aeruginosa* isolates tested with manuka honey and either tobramycin or colistin

Antibiotic	*Pseudomonas* *aeruginosa* reference number	Test method	MIC (µg/ml) antibiotic		FIC antibiotic
			Alone	With MH	
Colistin	867	Broth dilution	0.5	0.25	**0.5**
		E strip	0.38	0.19	**0.5**
Tobramycin	867	Broth dilution	128	128	**1**
		E strip	32	32	**1**
Colistin	1099	Broth dilution	16	4	**0.25**
		E strip	0.19	0.125	**0.66**
Tobramycin	1099	Broth dilution	16	0.5	**0.03**
		E strip	2	0.5	**0.25**

1 % (w/v) below MIC was used in synergy tests. The FIC values seen were consistent across the methods used

FIC values are in bold

*MH* manuka honey

**Table 3:** Susceptibility and FIC values of two clinical *B. cepacia* isolates to manuka honey and either tobramycin or colistin

Antibiotic	*Burkholderia* strain	Test method	MIC (µg/ml) antibiotic		FIC antibiotic
			Alone	With MH	
Colistin	*B. ambifaria*	Broth dilution	256	256	1
		E strip	256	256	1
Tobramycin	*B. ambifaria*	Broth dilution	256	256	1
		E strip	32	32	1
Colistin	*B. pyrrocinia*	Broth dilution	256	64	0.3
		E strip	256	64	0.3
Tobramycin	*B. pyrrocinia*	Broth dilution	32	32	1
		E strip	16	16	1

1 % (w/v) below MIC was used in synergy tests. The FIC values seen were consistent across the methods used

*MH* manuka honey

## Discussion

Tobramycin and colistin were chosen for the synergy studies here because they are licensed for use as inhaled antibiotics in treating chronic respiratory infections in cystic fibrosis. The use of inhaled drugs for treatment of respiratory infections is relatively new. Within the last 30 years, drugs that were originally designed for oral and intravenous application have been reformulated for inhalation; novel inhaled compounds such as gallium are also in development (Hofmann 2012). It is possible that honey combined with selected antibiotics could be developed into an inhalation compound for use in patients with respiratory infections. Combinations of antibiotics with non-antibiotic substances can enhance the efficacy of a number of currently used antibiotics forming syncretic combinations (Ejim et al. 2011; Garo et al. 2007). Additionally, combination therapy has been promoted as a strategy for reducing the emergence of antibiotic resistant bacteria and decreasing the likelihood that organisms exposed to them will survive. Already, manuka honey has been shown not to select for resistance (Cooper et al. 2010).

The findings above are consistent with data obtained in previous studies with raw manuka honey (Cooper et al. 2000, 2002), and it is important that the susceptibility of respiratory isolates to honey did not differ markedly from that of wound isolates. As discussed above, *P. aeruginosa* and *B. cepacia* are both potentially pathogenic bacteria with innate resistance to antibiotics. Incidence of multidrug resistance in these species other than respiratory conditions such as cystic fibrosis is not common, and in some cases, these infections can be cleared effectively. However, in complex and chronic infections, such as those seen in respiratory disorders, these bacteria can become highly tolerant to antibiotics and progressively more difficult to treat. As a consequence of this decreased susceptibility to individual antibiotics and to combinations of antibiotics, it has become ever more important to find new or complementary antibacterial agents to augment currently available drug regimes.

Compounding the problem of innate antibiotic resistance in these bacteria is their ability to establish microcolonies embedded in mucoid extracellular polymeric substances, otherwise known as biofilm. This is a particular problem in cases of cystic fibrosis. Such structures require the utilisation of high concentrations of antibiotic for inhibitory concentrations to be reached throughout the biofilm. Antibiotics delivered to cells within the biofilm at levels below the MIC due to inefficient diffusion through biofilms have been shown to induce growth of biofilm, specifically with tobramycin and *P. aeruginosa* (Linares et al. 2006). Use of sub-inhibitory antibiotics can also lead to increased resistance (Wu et al. 2009).

*P. aeruginosa* and *B. cepacia* are frequently associated with cystic fibrosis and hospital-acquired pneumonias which are associated with high morbidity and mortality. Poor control of these bacteria impacts on treatment times, increases risk of cross-infection and increases risk of long-term carriage in patients (Aloush et al. 2006; Chou 2006). Previously, studies have suggested that natural compounds combined with antibiotics could be a new strategy for combating infections and here it is apparent that honey could be a compound worthy of further investigation. Laboratory studies have also begun to report the susceptibility of biofilms of *P. aeruginosa* to manuka honey (Maddocks et al. 2013; Cooper et al. 2014), and the next logical step would be to evaluate the efficacy of combinations of honey and antibiotics on biofilms.

## Conclusions

The data presented here indicate that manuka honey at relatively low concentrations inhibits the growth of clinical cystic fibrosis isolates of both *P. aeruginosa* and *B. cepacia* and acts synergistically with two pertinent antibiotics. We believe that manuka honey may have a role to play in the management of cystic fibrosis patients with chronic respiratory infections, and in the future, we intend to investigate the activity of honey with a much larger range of bacteria and antibiotic combinations and to determine the susceptibilities of biofilms, as well as suspension cultures. Clinical studies will also be needed to determine the potential and efficacy of antibiotic and honey combinations for cystic fibrosis patients.
